# Reply to Comment on Shen H, *et al.* “Co-signaling receptors regulate T-cell plasticity and immune tolerance”. Frontiers in Bioscience-Landmark. 2019; 24: 96–132

**DOI:** 10.52586/4978

**Published:** 2021-10-30

**Authors:** Haitao Shen, Na Wu, Gayani Nanayakkara, Hangfei Fu, Qian Yang, William Y. Yang, Angus Li, Yu Sun, Charles Drummer, Candice Johnson, Ying Shao, Luqiao Wang, Keman Xu, Wenhui Hu, Marion Chan, Vincent Tam, Eric T. Choi, Hong Wang, Xiaofeng Yang

**Affiliations:** 1 Department of Emergency Medicine, Shengjing Hospital of China Medical University, 110004 Shenyang, Liaoning, China; 2 Centers for Metabolic Disease Research, Lewis Katz School of Medicine at Temple University, Philadelphia, PA 19140, USA; 3 Lewis Katz School of Medicine at Temple University, Philadelphia, PA 19140, USA; 4 Department of Endocrinology, Shengjing Hospital of China Medical University, 110004 Shenyang, Liaoning, China; 5 Department of Biomedical Engineering, Duke University, Durham, NC 27708, USA; 6 Department of Cardiovascular Medicine, the First Affiliated Hospital of Kunming Medical University, 650031 Kunming, Yunnan, China; 7 Department of Pathology, Lewis Katz School of Medicine at Temple University, Philadelphia, PA 19140, USA; 8 Department of Microbiology and Immunology, Lewis Katz School of Medicine at Temple University, Philadelphia, PA 19140, USA; 9 Department of Surgery, Lewis Katz School of Medicine at Temple University, Philadelphia, PA 19140, USA

As pointed out in this Comment [1], with a large amount of positive clinical experience of cancer immunotherapy with anti-PD1 or anti-CTLA4 monoclonal antibodies, co-signaling-related research has become a matter of intense interest. In our paper [2], we implemented comprehensive database mining to improve our understanding of co-signaling receptors, both co-stimulatory and co-inhibitory receptors. We are glad that in our paper you discerned some novel insights into co-signaling receptors that may potentially indicate new directions for therapeutic targeting in the treatment of cancers and inflammatory disorders. The results of this data mining exercise also provided some new insights into reverse signaling, T cell plasticity and the roles of endothelial cells in immune tolerance.

In response to your note on section 4.4, the point of this section was to clarify results suggesting that these tumors may have a better response to immunotherapy, not worse prognosis. However, we also need to point out that unfortunately there were some clerical errors in the paper that need to be specified to avoid misunderstandings when other researchers refer to this article:

1. The full name of CD112 in Table 1 should be “nectin cell adhesion molecule 2”, and the gene ID should be “NECTIN2”, while the binding receptors written as “TIGHT” should have been “TIGIT”;

2. The “Fig. 12A and Fig. 12B” in section 4.7 should be “Fig. 13A and Fig. 13B”;

3. The “Fig. 11” in section 4.6 should be “Fig. 12” mentioning that FLT4 RNAi regulates the expression of co-signaling receptors.

As mentioned in the Comment, we are also planning to implement experimental procedures to verify and support the results of our database mining and analysis. The experimental methods mentioned in your Comment, such as in vitro testing with patient-derived 3D organoids, have greatly inspired us, and made our experimental design more pertinent and clinically applicable.
